# Real-life data about obesity management

**DOI:** 10.1055/a-2515-1487

**Published:** 2025-01-29

**Authors:** Jacques Deviere

**Affiliations:** 1 26659 Gastroenterology, Erasme Hospital, Brussels, Belgium

**Keywords:** Epidemiology, Endoscopy Upper GI Tract, Obesity



Obesity and its metabolic consequences are the pandemic of our century. Bariatric surgery, the most effective and durable therapy for obesity, is only offered to a small minority of patients, mainly related to its perceived invasiveness and possible risks 1 . Endoscopic bariatric and metabolic therapies have been developed as an alternative primary procedure in selected patients with obesity or excess weight with comorbidities. As a restrictive approach, endoscopic gastric remodeling (EGR) is currently offered routinely using three different techniques that have been proven effective at short- and mid-term 2 3 4 . Anti-obesity medications (AOMs) represent another approach to this disease. Early drug use was limited due to their modest efficacy, the discomfort associated with their intake and/or their potential adverse events (AEs) 5 . Glucagon-like peptide 1 receptor agonists (GLP-1RAs), which mimic the action of enteric hormones that modify central appetite regulation and induce gastroparesis, have boosted the enthusiasm for AOMs, offering more than 15% weight loss at mid-term without significant AEs, except for nausea 6 . However, like other AOMs, they have to be prescribed over the long term to prevent weight regain, which very often is observed on discontinuation 7 . That obviously has an impact on treatment cost and patient compliance.

In this issue of the journal, Jirapinyo et al report on a large series of patients who had EGR procedure over a 5-year period in a referral bariatric center 8 . They compared the results of what they called combination therapy when patients (31%) had initiated AOMs treatment within 6 months before or after EGR, sequential therapy, when patients (35%) received AOMs more than 6 months before or after EGR, and monotherapy, when they (34%) had EGR alone. Furthermore, they differentiated two types of AOMs, namely GLP1RAs and all the others. At 1-year follow-up, the GLP1-RA + EGR combination group had the highest weight loss, followed by the EGR monotherapy group.

This conclusion is not surprising because GLP1RAs are currently the most effective drugs in terms of weight loss achieved after 1 year of therapy and EGR also has been proven effective for that period of follow-up. Other AOMs are less and less prescribed and this study further suggests that they are of limited efficacy and could become obsolete. The combination therapy, as defined by the authors, implies that, from the start of obesity management, a specific plan should be undertaken to integrate management with combined therapy that includes a GLP1RA initiated within 6 months of EGR performance. The poor results observed in patients who had sequential therapy also suggest that offering a treatment, waiting for its failure, and then offering another one is probably not the best approach.

The study by Jirapinyo et al represents real life, seen from a referral bariatric center that offers pharmacological, endoscopic, and surgical approaches. It provides information that is potentially useful for further investigations, but has several obvious biases. The major one is its retrospective design, which was based on a cohort of patients who effectively had EGR and which ignored those who had only pharmacotherapy and lifestyle education and who currently are the majority of patients with non-morbid obesity managed in bariatric centers. This design implies that sequential therapy could be associated with a lack of compliance and could explain the fact that it was less effective than EGR alone (whether prescribed before or after EGR).

These data, however, are of interest because the current major concern about GLP1RA therapy is weight regain after discontinuation, with obvious implications in terms of costs and potentially for safety. The major concern about endotherapy is lack of strong data about long-term maintenance of weight loss. AOMs are now highly effective, have been widely adopted worldwide, and could be combined with EGR to offer results that strongly compete with a surgical approach to morbid obesity. In patients with excess weight, comorbidities, and non-morbid obesity, it is highly probable that the first therapy initiated most often will be a GLP1RA, especially if they have diabetes (as shown in this study). Seen from an endoscopic perspective, it does not imply that EGR will have no remaining indication; to the contrary, this represents an opportunity to identify patients who will benefit the most from EGR over the mid-term and long-term. Instead of being seen as competitive, pharmacotherapy and endotherapy, when properly combined, appear to be complementary.

At the time of this publication, it is clear that we need further evidence to understand which combination of therapies will really help to manage this pandemic and lead to guidelines for further investigation. A previous study 9 showed that initiating a GLP1RA and performing EGR at the same time will only modestly improve total body weight loss (TBWL) compared with EGR alone (25% vs 21% TBWL at 1 year in a highly selected group). Giving all treatments to everybody is probably not the best solution and the results presented here suggest that combination therapy should include a GLP1RA, probably started initially (because, in any case, use of a GLP1RA is likely in the majority of centers, at least for non-morbid obesity). EGR would be the plan for patients who are compliant (that is, those who will follow the treatment and, thus, achieve significative weight loss), performed within 6 months after GLP1RA initiation, with the intent to interrupt GLP1RA therapy after a maximum of 1 year. Those patients, of course, should be followed by a dietician for up to 5 years after the start of therapy. This is obviously a subjective opinion about what should be studied in future trials in this area. Another approach might be to carefully select patients for EGR, perform the procedure, and plan to add GLP1RA therapy within 6 to 12 months in patients who regain weight. This, unfortunately, implies restarting long-term pharmacotherapy in these patients.

Whatever the choice, the real-life data presented in this paper suggest that further studies about obesity management should not be limited to comparisons of drug therapy or an endoscopic procedure with a placebo or a sham procedure, but should include combinations of the most effective nonsurgical approaches with long-term outcomes as primary endpoints.
